# *LINC02362* attenuates hepatocellular carcinoma progression through the *miR-516b-5p*/*SOSC2* axis

**DOI:** 10.18632/aging.203813

**Published:** 2022-01-06

**Authors:** Dezhi Li, Tao Zhou, Yaqin Li, Yanwei Xu, Xianyi Cheng, Junhui Chen, Wei V. Zheng

**Affiliations:** 1 Intervention and Cell Therapy Center, Peking University Shenzhen Hospital, Shenzhen 518036, Guangdong, China; 2 Department of Minimally Invasion Intervention, Peking University Shenzhen Hospital, Shenzhen 518036, Guangdong, China; 3 Department of Infectious Disease, Peking University Shenzhen Hospital, Shenzhen 518036, Guangdong, China

**Keywords:** LINC02362, hepatocellular carcinoma, miR-516b-5p, SOSC2, ceRNA

## Abstract

Hepatocellular carcinoma (HCC) is one of the most death-related cancers worldwide. Identifying cancer-associated genes and uncovering the vital molecular mechanisms of HCC progression contribute greatly to the prognosis and novel therapeutic strategies for HCC patients. Although lncRNAs have been proved to be critical modulators of various cellular processes, the functions of lncRNAs in HCC progression are just emerging. Here, we found that a long non-coding RNA (lncRNA) named *LINC02362*, whose biological effects have yet been unveiled in cancers, was associated with a better prognosis in patients with HCC. Gain-of-function analyses showed that *LINC02362* inhibited the survival, migration, invasion and epithelial-to-mesenchymal transition (EMT) of HCC cells. Moreover, *miR-516b-5p* was enriched as a target of *LINC02362*, which functioned as a sponge to regulate the endogenous levels of *miR-516b-5p*. Furthermore, we confirmed that *SOSC2* served as a downstream target gene which was negatively controlled by *miR-516b-5p*. Importantly, a series of rescue experiments indicated that the tumor-suppressive effects of *LINC02362* were achieved through the modulation of the *miR-516b-5p*/*SOSC2* axis. In summary, we identified *LINC02362* as a candidate tumor-inhibitory lncRNA that might serve as a biomarker for the prognosis of HCC and a promising therapeutic agent for patients with HCC.

## INTRODUCTION

Hepatocellular carcinoma (HCC) is the fourth leading cause of cancer-related deaths [1]. HCC is usually initiated by hepatitis B (HBV) infection and chronic liver diseases, resulting in the genetic aberrations of key driver genes, such as *p53* and *CTNNB* [2]. Although systematic treatments have significantly increased the five-year survival rate of patients with HCC, exploring the underlying molecular mechanisms of HCC is highly required for aiding to the development of more effective therapeutic strategies.

Less than 2% of the human genome can be transcribed into protein-coding mRNAs, while more than 75% is actively transcribed into non-coding RNAs, such as microRNAs (miRNAs), long non-coding RNAs (lncRNAs), and circular RNAs (circRNAs) [3]. LncRNAs are a family of non-coding RNAs that are longer than 200 nucleotides (nts) in length and unable to be translated into proteins [4, 5]. LncRNAs have been found to affect multitudes of cellular processes via numerous mechanisms [6]. Nuclear lncRNAs modulate the transcription of target genes by interacting with transcription factors or chromatin modifiers. In addition, mRNA splicing and chromatin interaction were also proved to be mediated by lncRNAs that are localized in the nucleus [7]. When localized in the cytosol, lncRNAs have been shown to directly bind to mRNAs or proteins through base-pairing or specific secondary structures, leading to the stability or/and activity alterations of these macromolecules [6]. Another well-known mechanism of cytoplasmic lncRNAs is functioning as sponges for miRNAs [8].

MiRNAs are 22-nt small non-coding RNAs that enhance the degradation or/and translation of mRNAs by binding to the 3’ untranslated regions (UTRs) [9]. Thus, through sponging miRNAs, lncRNAs are capable to indirectly enhance the levels of target mRNAs. Multitudes of lncRNAs such as *lnc-ATB* [10], *lncRNA-LALR1* [11], and *lncHAND2* [12] have been found to aberrantly expressed and serve as modulators during the progression of HCC. Suppressors of cytokine signalling 2 (*SOCS2*) has been shown to correlate with various inflammatory diseases and cancer [13]. In HCC patients, low expression of *SOCS2* is associated with advanced TNM staging and is a promising prognostic marker [14].

Although lncRNAs such as *MIAT* and *PCNAP1* have been shown to promote the HCC progression, the functions and underlying mechanisms of lncRNAs in HCC still need to be explored [15, 16]. As long intergenic non-protein coding RNA 2362 (*LINC02362*) is a lncRNA whose biological functions are ill-investigated, we aimed to check whether *LINC02362* is involved in the progression of HCC. Through online datamining, we observed that *LINC02362* was lower expressed in HCC patient samples and was correlated with favorable outcomes of HCC patients. Depletion of *LINC02362* in HCC cells resulted in the enhanced survival, migratory and invasive properties as well as the induction of epithelial-to-mesenchymal transition (EMT). In addition, *miR-516b-5p* was enriched as a target miRNA of *LINC02362*. Furthermore, *LINC02362* promoted the expression of *SOSC2* via sponging *miR-516b-5p*, leading to the alleviation of HCC cell survival, migration, invasion and EMT. In summary, we shed light on the mechanism by which *LINC02362* exerts its novel negative regulatory functions during HCC progression, which may provide HCC treatments with a new therapeutic agent.

## RESULTS

### *LINC02362* is associated with a better prognosis of HCC patients

**Figure 1:**
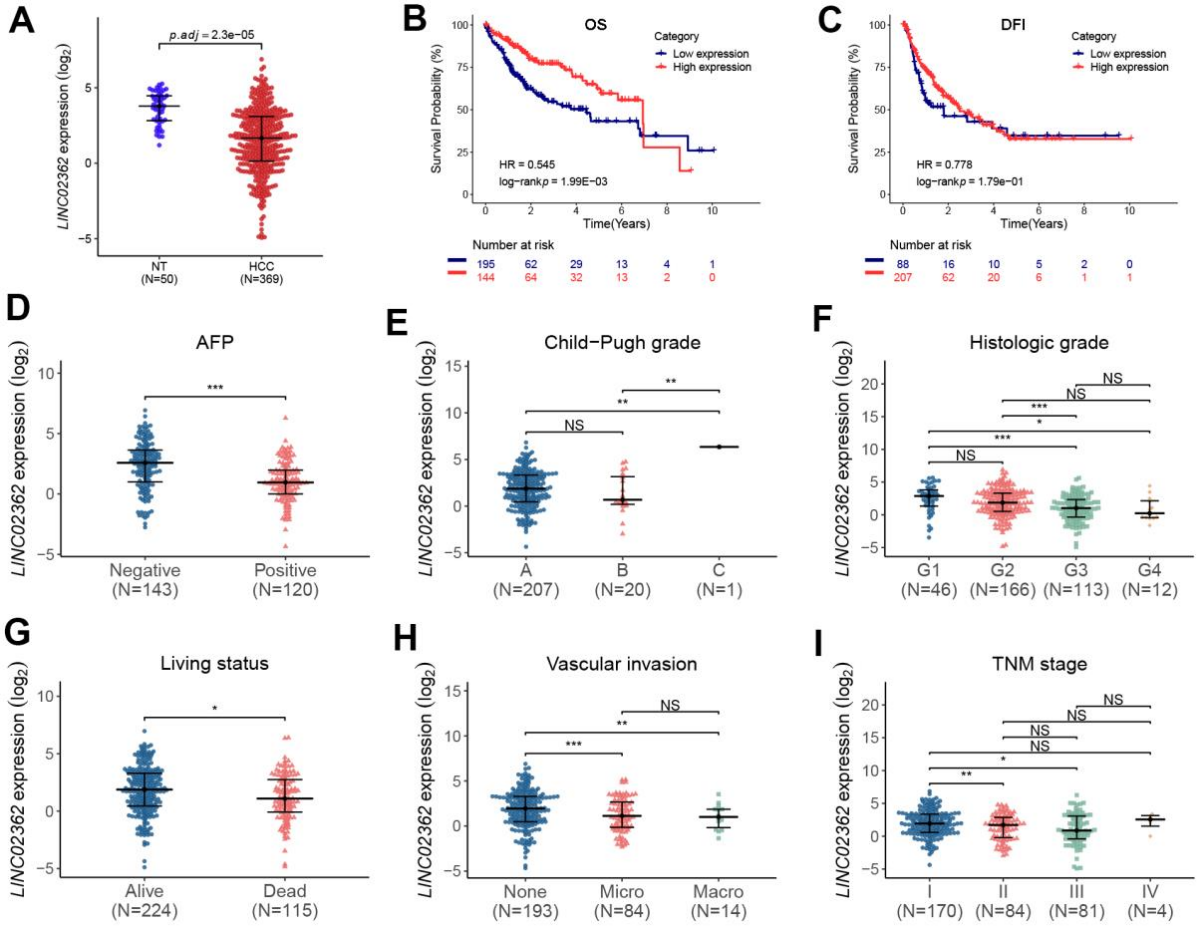
***LINC02362* is correlated with a favorable prognosis of HCC patients.** (**A**) Differential analyses of *LINC02362* levels in non-tumor (NT; n=50) or HCC tissues (n=369). (**B**, **C**) Kaplan-Meier plots showing the overall survival (OS; **B**) or disease-free interval (DFI; **C**) of HCC patients stratified by *LINC02362* levels. (**D**–**I**) The analyses of expression of *LINC02362* in HCC patients with serum alpha feto-protein (AFP)-negative or positive (**D**), different Child-Pugh grades (**E**), histological grades (**F**), living status (**G**), vascular invasion (**H**) or TNM stages (**I**). *0.01 < *P* < 0.05, **0.001 < *P* < 0.01, ***0.0001 < *P* < 0.001. NS, not significant.

As *LINC02362* is an annotated lncRNA whose biological roles have not been well studied, it is worth investigating whether *LINC02362* is involved in the progression of HCC. To this end, we checked the levels of *LINC02362* in non-tumor and HCC samples. Interestingly, we observed that *LINC02362* was expressed at significantly lower levels in HCC samples than those in non-tumor liver samples (Figure 1A). Next, we performed data analysis in the TCGA-LIHC database which includes the clinical parameters of HCC patients. As shown in the Kaplan-Meier plots in Figure 1B, 1C, low expression of *LINC02362* was correlated with a poor prognosis in terms of overall survival (OS) and disease-free interval (DFI). It is of note that, between the two groups, i.e. *LINC02362*-low and *LINC02362*-high, the OS trend was reversed after six years and there was almost no significant difference in terms of DFI (Figure 1B, 1C). We supposed that many patients might die before six years or they developed metastases at this time point, resulting in only a few patients (with seven patients in Figure 1B and four patients in Figure 1C) were still alive after this time point and the trend after six years may not be representative due to the low amount of patients. In addition, *LINC02362* was less expressed in serum alpha-fetoprotein (AFP)-positive HCC patients whose prognosis is more favorable (Figure 1D) [17]. Nevertheless, we noticed that *LINC02362* was higher expressed in stage C than in stages A and B in terms of Child-Pugh classification, which was not as expected and will be discussed in the discussion section (Figure 1E). Furthermore, we found that *LINC02362* was significantly decreased in the later histologic or TNM grades in comparison with the early benign grade G1 or stage I, respectively (Figure 1F, 1I). Moreover, *LINC02362* was downregulated in dead HCC patients than that in survivors (Figure 1G). Since vascular invasion is a hallmark of HCC progression [18], we analyzed *LINC02362* levels in HCC patients with or without vascular invasion. Surprisingly, *LINC02362* was expressed at lower levels in patients with microvascular or macrovascular invasion than that in those without vascular invasion (Figure 1H). Lower *LINC02362* level was observed in TNM stage II and III than in their corresponding controls. Correlation between *LINC02362* expression and clinicopathological variables was summarized in Supplementary Table 3. Collectively, *LINC02362* is correlated with a better prognosis in HCC patients.

### *LINC02362* mitigates HCC cell survival, migration, invasion and EMT

**Figure 2:**
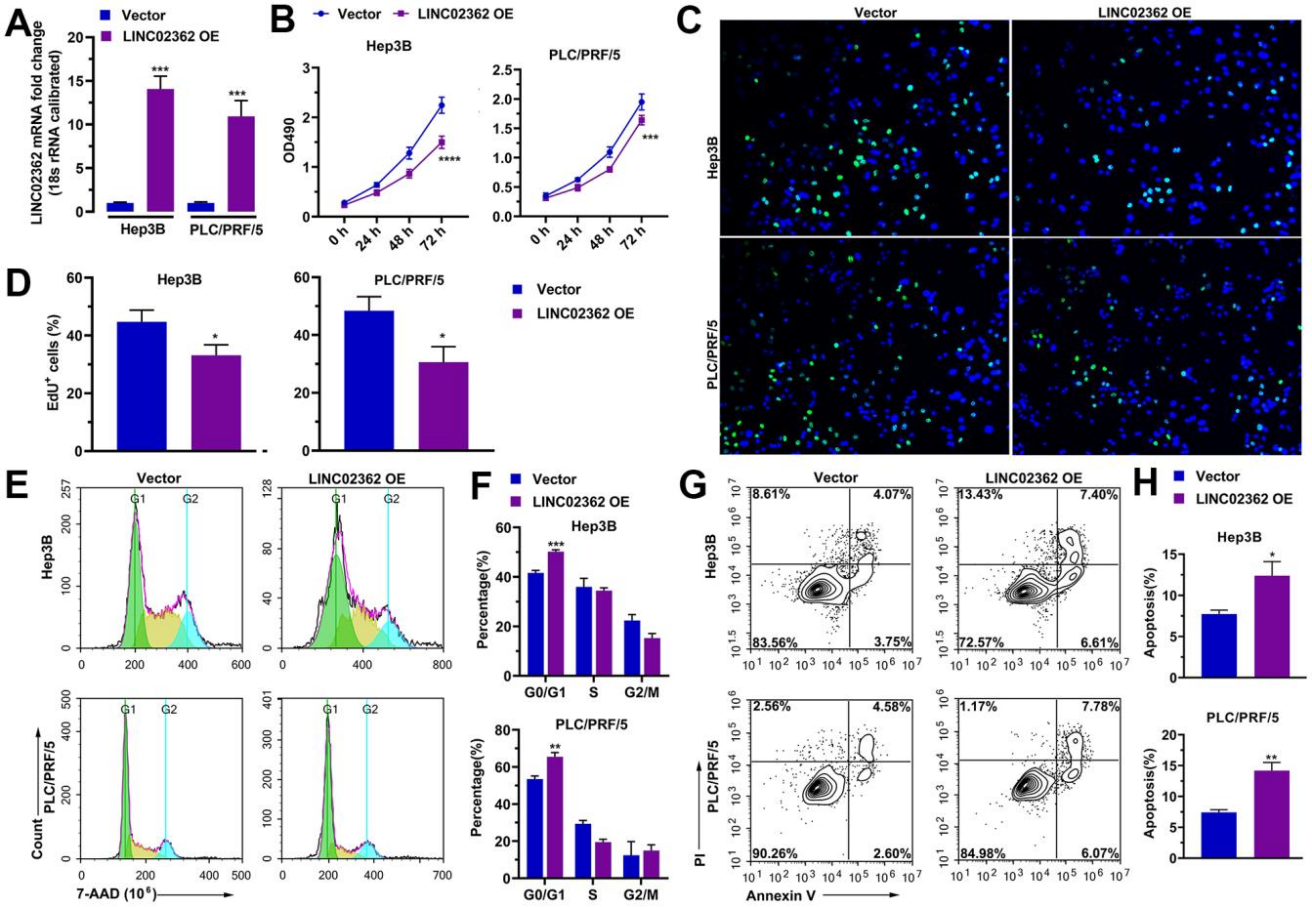
***LINC02362* suppresses HCC cell survival.** (**A**) RT-qPCR quantification (n=3) of *LINC02362* expression in Hep3B or PLC/PRF/5 cells with *LINC02362* ectopic expression. (**B**) MTT assay (n=3) for measuring the proliferative abilities of HCC cells with *LINC02362* overexpression. (**C**, **D**) EdU labeling to detect the percentage of dividing cells in *LINC02362*-overexpressing HCC cells and the corresponding quantification (**D**; n=3). (**E**, **F**) Measurement (**E**) and quantification (**F**; n=3) of cell cycle of HCC cells by flow cytometry. (**G**, **H**) Detection (**G**) and quantification (**H**; n=3) of apoptotic cells in HCC cells overexpressing *LINC02362*. *0.01 < *P* < 0.05, **0.001 < *P* < 0.01, ***0.0001 < *P* < 0.001, ****P < 0.0001.

**Figure 3:**
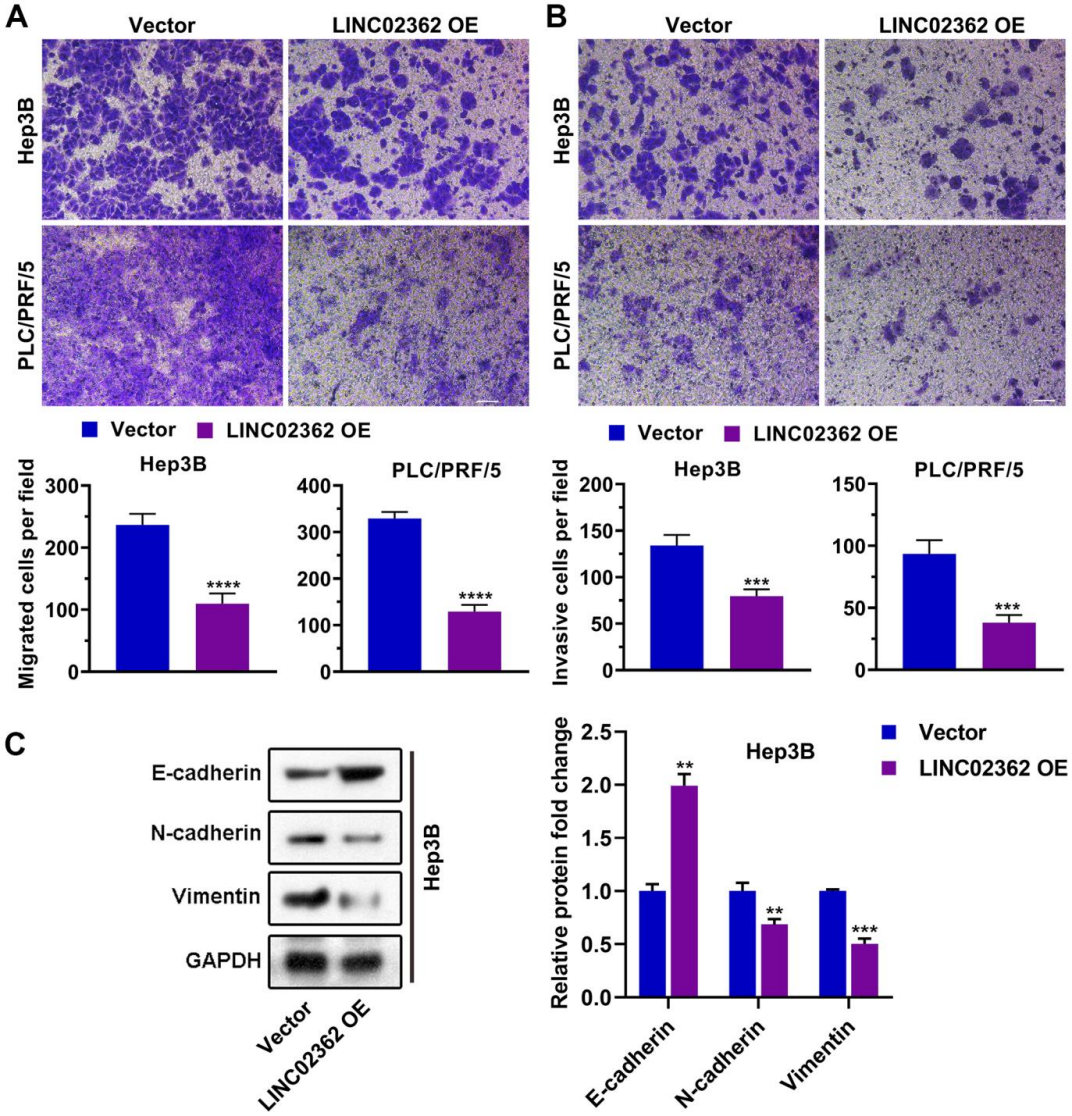
***LINC02362* inhibits HCC cell migration, invasion and EMT.** (**A**) Transwell assays to test the effects of *LINC02362* overexpression on HCC cell migration. Representative pictures (upper) and quantification (lower; n=3) are shown. (**B**) Transwell assays to test the effects of *LINC02362* overexpression on HCC cell invasion. Representative pictures (upper) and quantification (lower; n=3) are shown. (**C**) Representative images (left) and quantification (right; n=3) of western blotting analysis for detecting the levels of EMT markers in Hep3B cells. **0.001 < *P* < 0.01, ***0.0001 < *P* < 0.001, ****P < 0.0001.

Based on the fact that *LINC02362* was associated with better outcomes in patients with HCC, we hypothesized that *LINC02362* might function as a tumor-suppressive lncRNA. To test our assumption, *LINC02362* was ectopically expressed in Hep3B and PLC/PRF/5 cells, both of which are commonly used cell lines for HCC research (Figure 2A). The MTT assay showed that overexpression of *LINC02362* mitigated the proliferation of these two HCC cell lines (Figure 2B). This conclusion was further confirmed by the EdU labeling experiments (Figure 2C, 2D). We then investigated whether the cell cycle was altered upon *LINC02362* misexpression. As expected, overexpressing *LINC02362* potentiated the cell cycle arrest in HCC cell lines (Figure 2E, 2F). As apoptosis might contribute to the change of cell survival, we evaluated the apoptotic ability of HCC cells. We found that *LINC02362* ectopic expression enhanced the apoptosis of HCC cells (Figure 2G, 2H). Moreover, we observed that *LINC02362* suppressed the migratory and invasive abilities of HCC cells (Figure 3A, 3B). Since the gain of cell migratory and cell invasive abilities is tightly correlate with the induction of EMT [19], we detected the changes of EMT markers in Hep3B cells. Upon the ectopic expression of *LINC02362*, the levels of epithelial marker E-cadherin was enhanced while the levels of two mesenchymal markers N-cadherin and Vimentin were significantly decreased (Figure 3C), indicating the EMT process was attenuated by *LINC02362*. Taken together, overexpression of *LINC02362* decreases the survival abilities by inhibiting cell proliferation, improving cell apoptosis, and alleviating the migration, invasion and EMT in HCC cells, suggesting that *LINC02362* is a lncRNA that attenuates HCC progression.

### *miR-516b-5p* is a target miRNA of *LINC02362*

**Figure 4:**
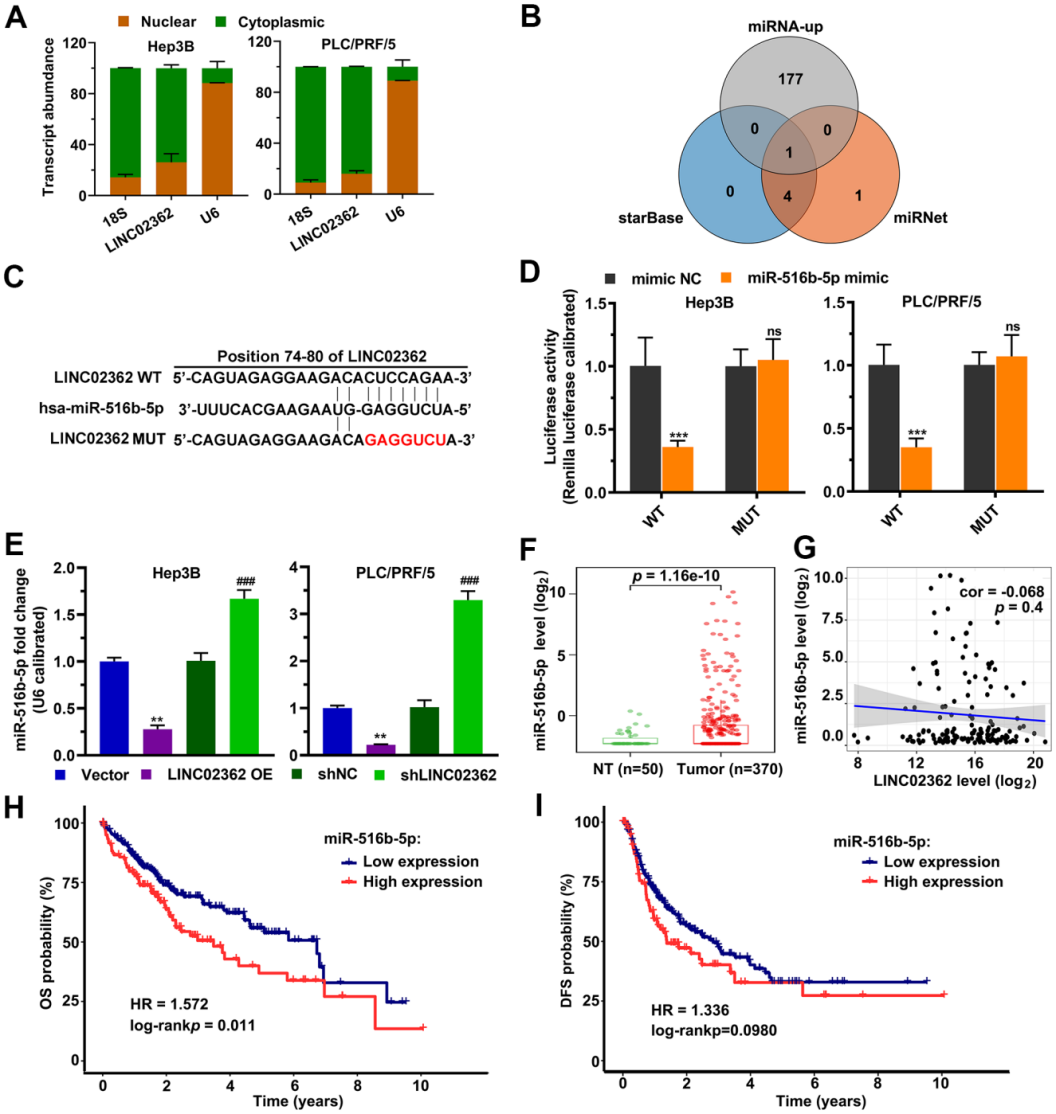
***LINC02362* functions as a sponge for *miR-516b-5p*.** (**A**) Subcellular fractionation (n=3) for quantifying the localization of *LINC02362* in HCC cells. (**B**) Venn diagram showing the overlap between the indicated three databases. (**C**) Schematic plot indicating the putative binding site between *LINC02362* and *miR-516b-5p* and the sequences upon mutagenesis. (**D**) Dual luciferase assays (n=3) for checking the effects of *miR-516b-5p* on the indicated 3’UTR constructs. (**E**) RT-qPCR detection (n=3) of the *miR-516b-5p* expression upon misexpression of *LINC02362*. (**F**) Datamining to check the levels of *miR-516b-5p* in non-tumor (NT; n=50) or HCC tissues (n=370). (**G**) Scatter plot showing the correlation between *LINC02362* and *miR-516b-5p*. (**H**, **I**) Kaplan-Meier plots showing the overall survival (OS, **H**) or disease-free survival (DFS, **I**) of HCC patients stratified by *miR-516b-5p* levels. **0.001 < *P* < 0.01, *** or ### 0.0001 < *P* < 0.001. NS, not significant.

Since the biological function of a lncRNA is highly dependent on its localization in cells [20], we first checked where *LINC02362* is localized. Subcellular fractionation experiments, in which cytosolic RNA *18S* and nuclear RNA *U6* were used as positive controls, showed that *LINC02362* was mainly localized in the cytoplasm (Figure 4A), which was confirmed by the Fluorescent *in situ* Hybridization (FISH; Supplementary Figure 1) and *in silico* prediction data from an online database (Supplementary Figure 2). Because a common mechanism of cytoplasmic lncRNAs is functioning as competitive endogenous RNAs (ceRNAs) to regulate the levels of target miRNAs [21], we checked the potential miRNA targets of *LINC02362* by *in silico* prediction. After overlapping the miRNAs upregulated in the TCGA-LIHC dataset with miRNAs that were predicted as *LINC02362* candidate targets in two databases, we enriched *miR-516b-5p* as the only miRNA in the intersection of these three datasets (Figure 4B). As lncRNA-miRNA interaction leads to the downregulation of both lncRNA and miRNA, we then performed a luciferase reporter assay to test the effects of *miR-516b-5p* on *LINC02362*. Relative luciferase activity indicated that *miR-516b-5p* overexpression caused a decrease in the reporter consisting of the interacting fragment of *LINC02362*, while mutating the nucleotides responsible for *miR-516b-5p* binding on *LINC02362* released the inhibitory effect (Figure 4C, 4D). In addition, ectopic expression of *LINC02362* suppressed *miR-516b-5p* levels while depletion of *LINC02362* enhanced the *miR-516b-5p* expression (Figure 4E). Furthermore, we showed that *miR-516b-5p* was highly expressed in HCC tumor samples compared with the non-tumor specimens (Figure 4F). In addition, although there was an inverse correlation between *LINC02362* and *miR-516b-5p*, the association was insignificant (Figure 4G). However, we did observed that high *miR-516b-5p* levels were correlated with poor prognosis of HCC patients (Figure 4H, 4I). Taken together, we validated *miR-516b-5p* as a downstream target of *LINC02362*.

### *LINC02362* sponges *miR-516b-5p* to mitigate HCC progression

**Figure 5:**
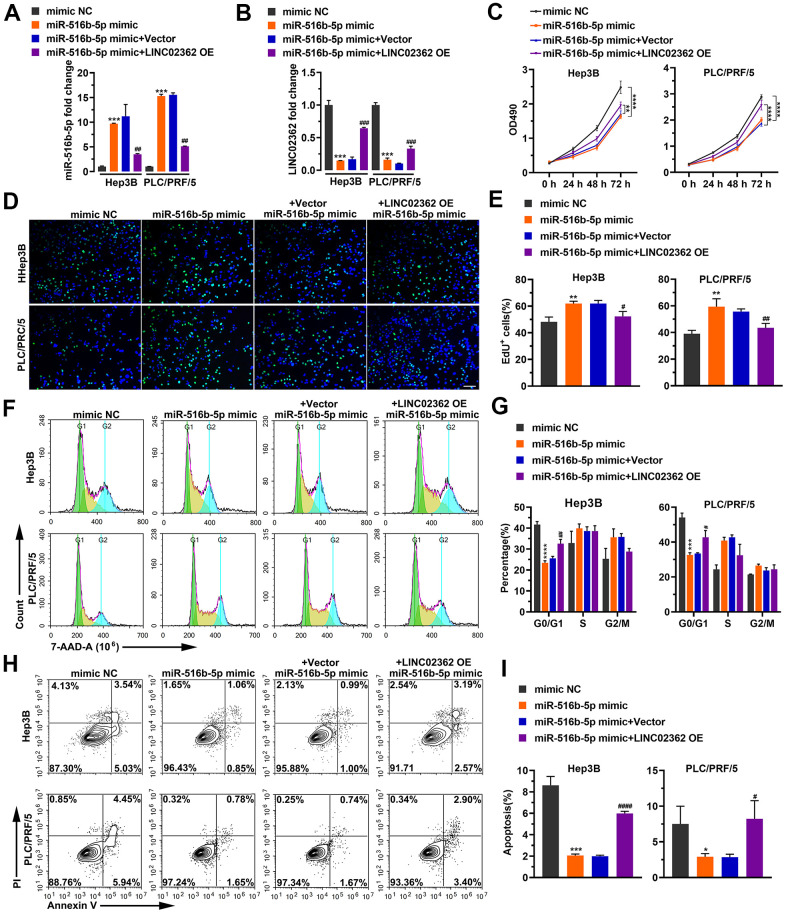
***LINC02362* sponges *miR-516b-5p* to mitigate the enhancement of HCC cell survival.** (**A**, **B**) RT-qPCR quantification of *miR-516b-5p* (**A**) and *LINC02362* (**B**) expression in Hep3B and PLC/PRF/5 cells (n=3). (**C**) MTT assay (n=3) for measuring the proliferative abilities of HCC cells overexpressing *miR-516b-5p* and/or *LINC02362*. (**D**, **E**) EdU labeling to detect the percentage of dividing cells in *LINC02362* and *miR-516b-5p*-overexpressing HCC cells (**D**) and the corresponding quantification (**E**; n=3). Bar=100μm. (**F**, **G**) Measurement (**F**) and quantification (**G**; n=3) of the cell cycle in HCC cells by flow cytometry. (**H**, **I**) Detection (**H**) and quantification (**I**; n=3) of apoptotic cells in HCC cells overexpressing *LINC02362* and *miR-516b-5p*. * or # 0.01 < *P* < 0.05, ** or ## 0.001 < *P* < 0.01, *** or ### 0.0001 < *P* < 0.001, **** or #### *P* < 0.0001.

**Figure 6:**
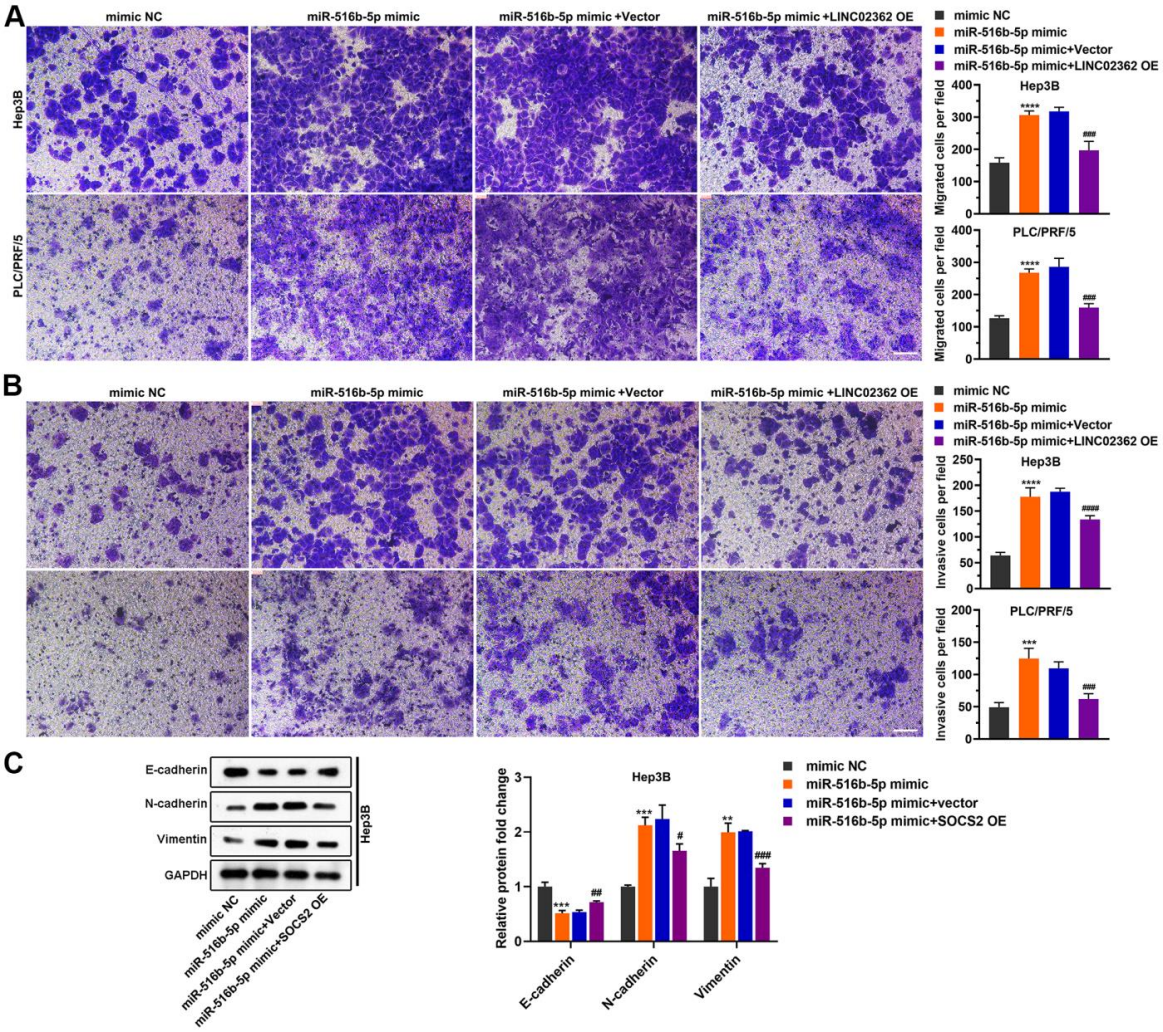
***LINC02362* sponges *miR-516b-5p* to attenuate the augment of HCC cell migration and invasion.** (**A**) Transwell assays to test the effects of *LINC02362* and *miR-516b-5p* overexpression on HCC cell migration. Representative images (left) and quantification (right; n=3) are shown. (**B**) Transwell assays to test the effects of *LINC02362* and *miR-516b-5p* overexpression on HCC cell invasion. Representative images (left) and quantification (right; n=3) are shown. (**C**) Representative images (left) and quantification (right; n=3) of western blotting analysis for detecting the levels of EMT markers in Hep3B cells. # 0.01 < *P* < 0.05, ** or ## 0.001 < *P* < 0.01, *** or ### 0.0001 < *P* < 0.001, **** or #### *P* < 0.0001.

Next, we asked whether the biological effects of *miR-516b-5p* on HCC cells can be attenuated by *LINC02362* as we showed that these two RNAs interact with each other (Figure 4C, 4D). First, RT-qPCR results suggested that the levels of exogenously expressed *miR-516b-5p* could be inhibited upon *LINC02362* ectopic expression (Figure 5A). Vice versa, endogenous or exogenous *LINC02362* was suppressed by the overexpression of *miR-516b-5p* (Figure 5B), suggesting that these two RNA molecules modulate the levels of each other in a negative manner. Furthermore, MTT data showed that the proliferation-supporting effect imposed by *miR-516b-5p* was significantly mitigated upon the ectopic expression of *LINC02362* (Figure 5C). Similarly, the EdU signal and cell cycle progression improved by *miR-516b-5p* was attenuated by *LINC02362* (Figure 5D–5G). Moreover, the cell apoptosis decreased by *miR-516b-5p* could be significantly alleviated by *LINC02362* (Figure 5H, 5I). Furthermore, *LINC02362* rescued the migration- and invasion-enhancing effects as well as the induction of EMT imposed by *miR-516b-5p* (Figure 6A–6C). Collectively, our data demonstrated that *LINC02362* sponges *miR-516b-5p* to mitigate the tumor-promoting effects exerted by *miR-516b-5p*.

### The effects of *LINC02362* on HCC cells are dependent on *miR-516b-5p*

**Figure 7:**
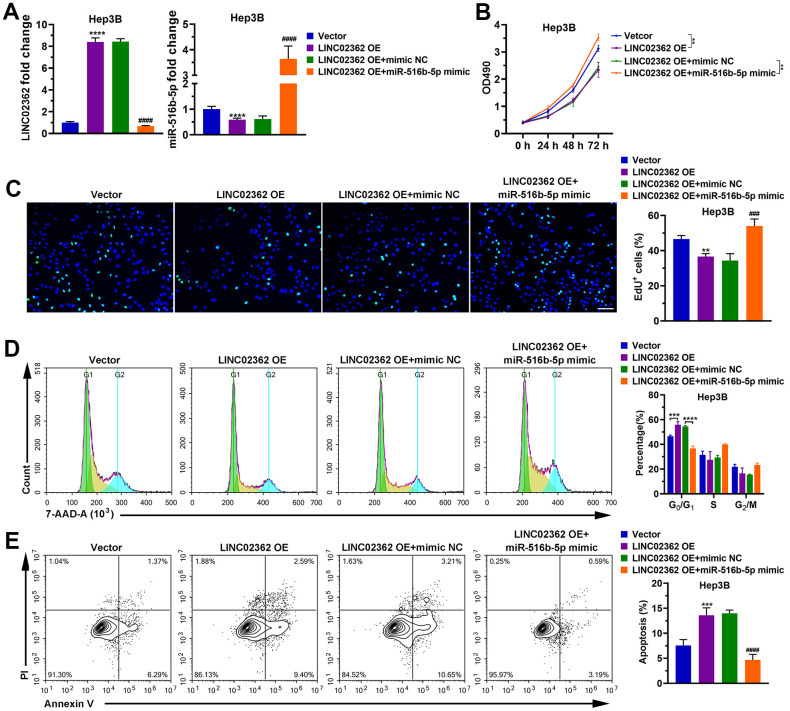
***miR-516b-5p* is critical for the effects of *LINC02362* on HCC cell survival.** (**A**) RT-qPCR quantification of *miR-516b-5p* (left) and *LINC02362* (right) expression in Hep3B and PLC/PRF/5 cells (n=3). (**B**) MTT assay (n=3) for measuring the proliferative abilities of HCC cells overexpressing *miR-516b-5p* and/or *LINC02362*. (**C**) EdU labeling to detect the percentage of dividing cells in *LINC02362* and *miR-516b-5p*-overexpressing HCC cells (left) and the corresponding quantification (right; n=3). Bar=100μm. (**D**) Measurement (left) and quantification (right; n=3) of the cell cycle in HCC cells by flow cytometry. (**E**) Detection (left) and quantification (right; n=3) of apoptotic cells in HCC cells overexpressing *LINC02362* and *miR-516b-5p*. ** 0.001 < *P* < 0.01, *** or ### 0.0001 < *P* < 0.001, **** or #### *P* < 0.0001.

**Figure 8:**
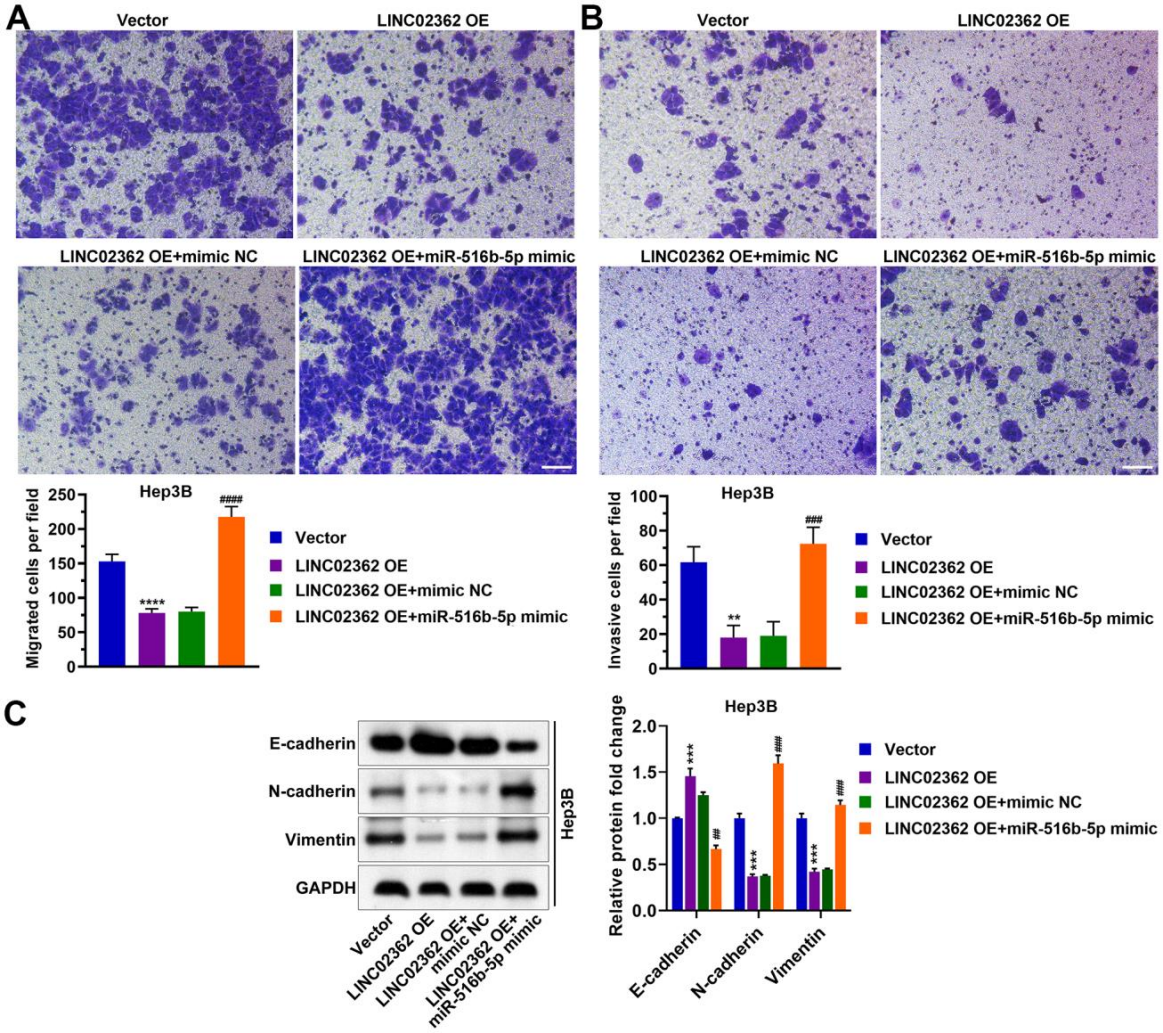
***miR-516b-5p* is pivotal for the effects of *LINC02362* on the migration, invasion and EMT of HCC cells.** (**A**) Transwell assays to test the effects of *LINC02362* and *miR-516b-5p* overexpression on HCC cell migration. Representative images (left) and quantification (right; n=3) are shown. (**B**) Transwell assays to test the effects of *LINC02362* and *miR-516b-5p* overexpression on HCC cell invasion. Representative images (left) and quantification (right; n=3) are shown. (**C**) Representative images (left) and quantification (right; n=3) of western blotting analysis for detecting the levels of EMT markers in Hep3B cells. ** or ## 0.001 < *P* < 0.01, *** or ### 0.0001 < *P* < 0.001, **** *P* < 0.0001.

To examine whether *miR-516b-5p* is critical for the effects of *LINC02362* on HCC cells, we ectopically expressed *miR-516b-5p* in HCC cells with *LINC02362* overexpression. Interestingly, we observed that the inhibition of cell proliferation, migration, invasion and EMT as well as the promotion of cell cycle arrest and apoptosis imposed by *LINC02362* were restored when *miR-516b-5p* was re-expressed (Figures 7, 8). Collectively, we confirmed that the tumor-suppressive roles of *LINC02362* on HCC cells is highly dependent on the inhibition of *miR-516b-5p*.

### *SOCS2* is a downstream gene of *miR-516b-5p*

**Figure 9:**
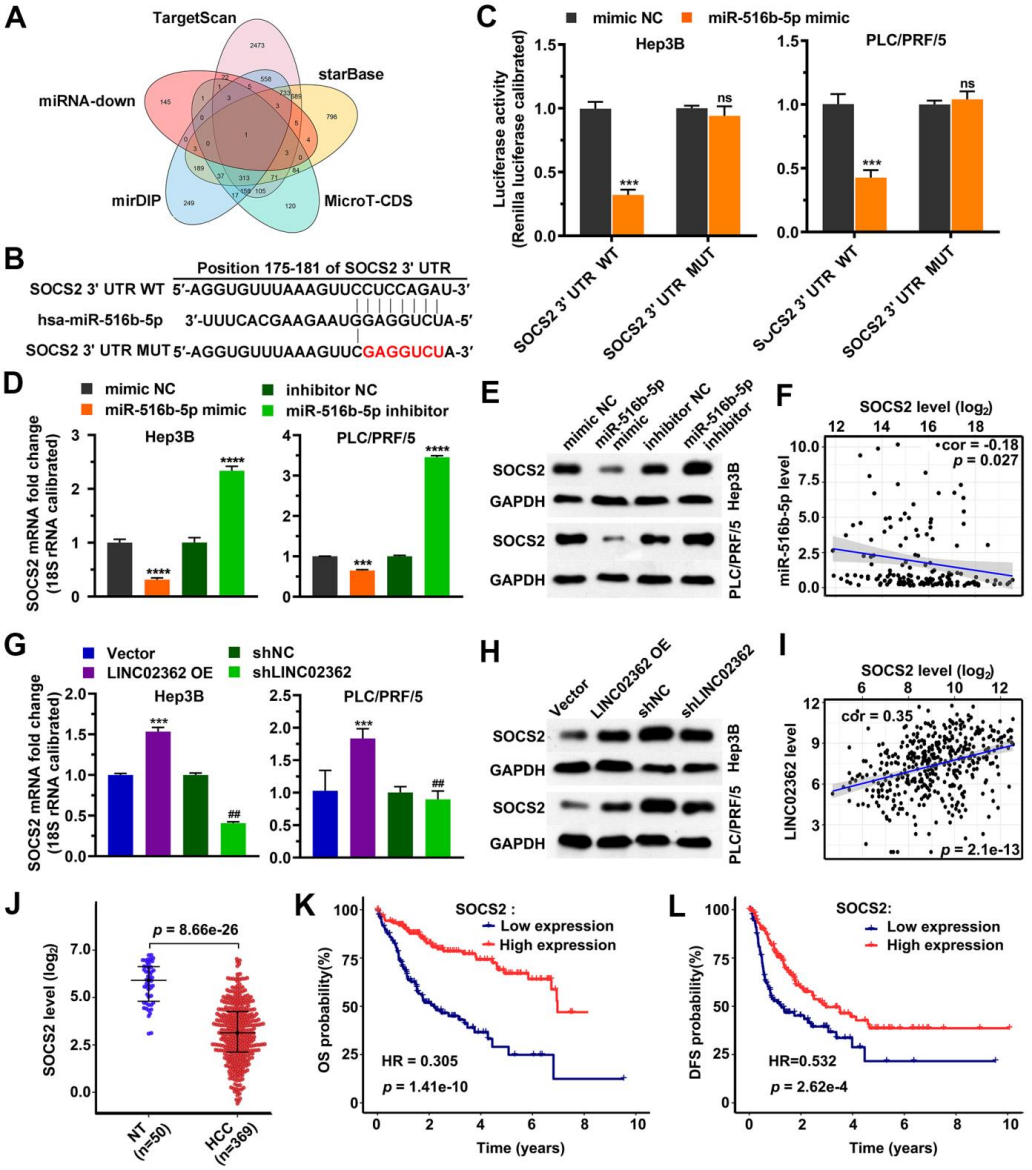
***SOCS2* is a downstream target gene of *miR-516b-5p*.** (**A**) Venn diagram indicating the overlap between the indicated 5 databases. (**B**) Schematic plot expressing the putative binding site between *miR-516b-5p* and *SOCS2* and the sequences upon mutagenesis. (**C**) Dual luciferase assays (n=3) for checking the effects of *miR-516b-5p* on the indicated 3’UTR constructs. (**D**, **E**) RT-qPCR (**D**) and western blotting (**E**) detection (n=3) of the *SOCS2* expression upon misexpression of *miR-516b-5p*. (**F**) Scatter plot showing the correlation between *miR-516b-5p* and *SOCS2*. (**G**, **H**) RT-qPCR (**G**) and western blotting (**H**) detection (n=3) of the *SOCS2* expression upon misexpression of *LINC02362*. (**I**) Scatter plot showing the correlation between *LINC02362* and *SOCS2*. (**J**) Data mining to check the levels of *SOCS2* in non-tumor (NT; n=50) or HCC tissues (n=369). (**K**, **L**) Kaplan-Meier plots showing the overall survival (OS, **K**) or disease-free survival (DFS, **L**) of HCC patients stratified by *SOCS* expression. ## 0.001 < *P* < 0.01, *** 0.0001 < *P* < 0.001, **** *P* < 0.0001. NS, not significant.

Since miRNAs exert their functions by binding to and inhibiting the stability of specific mRNAs [22], we then predicted candidate target mRNAs of *miR-516b-5p*. Upon performing overlap analysis based on five databases, *SOCS2* was enriched as the sole potential hit downstream *of miR-516b-5p* (Figure 9A). The following reporter assay indicated that *miR-516b-5p* suppressed the activity of wild-type but not the mutant *SOCS2* 3’UTR element (Figure 9B, 9C). RT-qPCR and western blotting analysis further validated the inhibition of *miR-516b-5p* on *SOCS2* at both mRNA and protein levels (Figure 9D, 9E). Moreover, data from patients with HCC revealed a negative correlation between *miR-516b-5p* and *SOCS2* levels, which supported the reversely regulatory relationship between these two molecules (Figure 9F). Since we have proved that *miR-516b-5p* is a downstream effector of *LINC02362* (Figures 4, 5), the expression of *SOCS2*, a target mRNA of *miR-516b-5p*, might be indirectly regulated by *LINC02362*. As expected, both mRNA and protein of *SOCS2* were upregulated by *LINC02362* ectopic expression and downregulated by *LINC02362* depletion (Figure 9G, 9H). The positive correlation between *LINC02362* and *SOCS2* was confirmed in the HCC patients as well (Figure 9I). Furthermore, the data also showed that *SOCS2* mRNA levels were decreased in HCC samples compared with non-tumor samples (Figure 9J). Interestingly, low levels of *SOCS2* were shown to be associated with worse outcomes in HCC patients (Figure 9K, 9L). In summary, we identified *SOCS2* as a direct target gene of *miR-516b-5p* and as an indirect target gene of *LINC02362*.

### *SOCS2* contributes to the tumor-suppressive function of *LINC02362*

**Figure 10:**
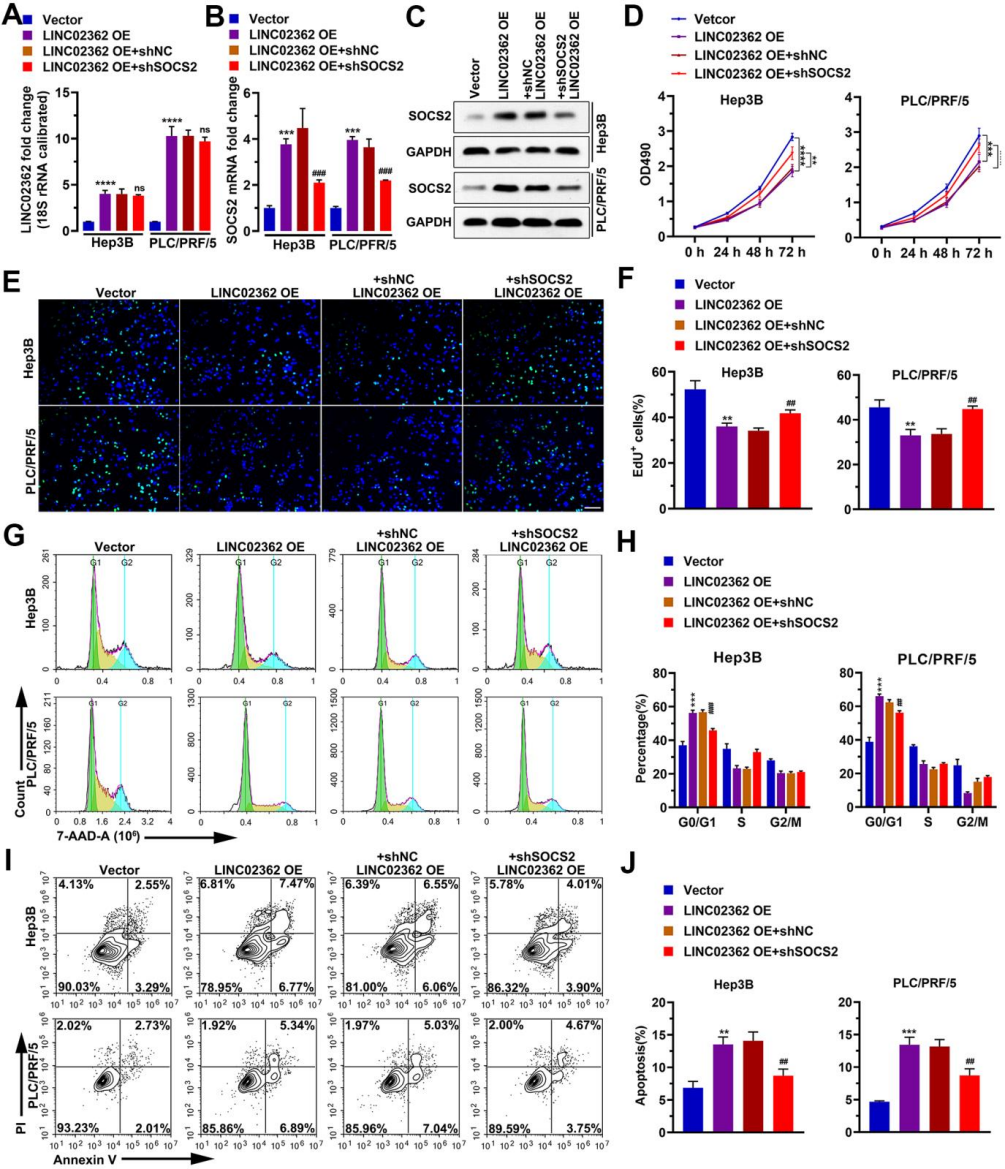
**SOCS2 mediates the inhibitory effects of *LINC02362* on HCC cell survival.** (**A**, **B**) RT-qPCR quantification (n=3) of *LINC02362* (**A**) and *SOCS2* (**B**) expression in Hep3B and PLC/PRF/5 cells. (**C**) Quantification of SOCS2 levels by western blotting (n=3) with *LINC02362* and SOCS2 misexpression. (**D**) MTT assay (n=3) for measuring the proliferative abilities of HCC cells with *LINC02362* and SOCS2 misexpression. (**E**, **F**) EdU labeling to detect the percentage of dividing cells in *LINC02362* and SOCS2 misexpressing HCC cells (**E**) and the corresponding quantification (**F**; n=3). Bar=100μm. (**G**, **H**) Measurement (**G**) and quantification (**H**; n=3) of the cell cycle in HCC cells by flow cytometry. (**I**, **J**) Detection (**I**) and quantification (**J**; n=3) of apoptotic cells in HCC cells misexpressing *LINC02362* and SOCS2. # 0.01 < *P* < 0.05, ** or ## 0.001 < *P* < 0.01, *** or ### 0.0001 < *P* < 0.001, **** *P* < 0.0001. NS, not significant.

**Figure 11:**
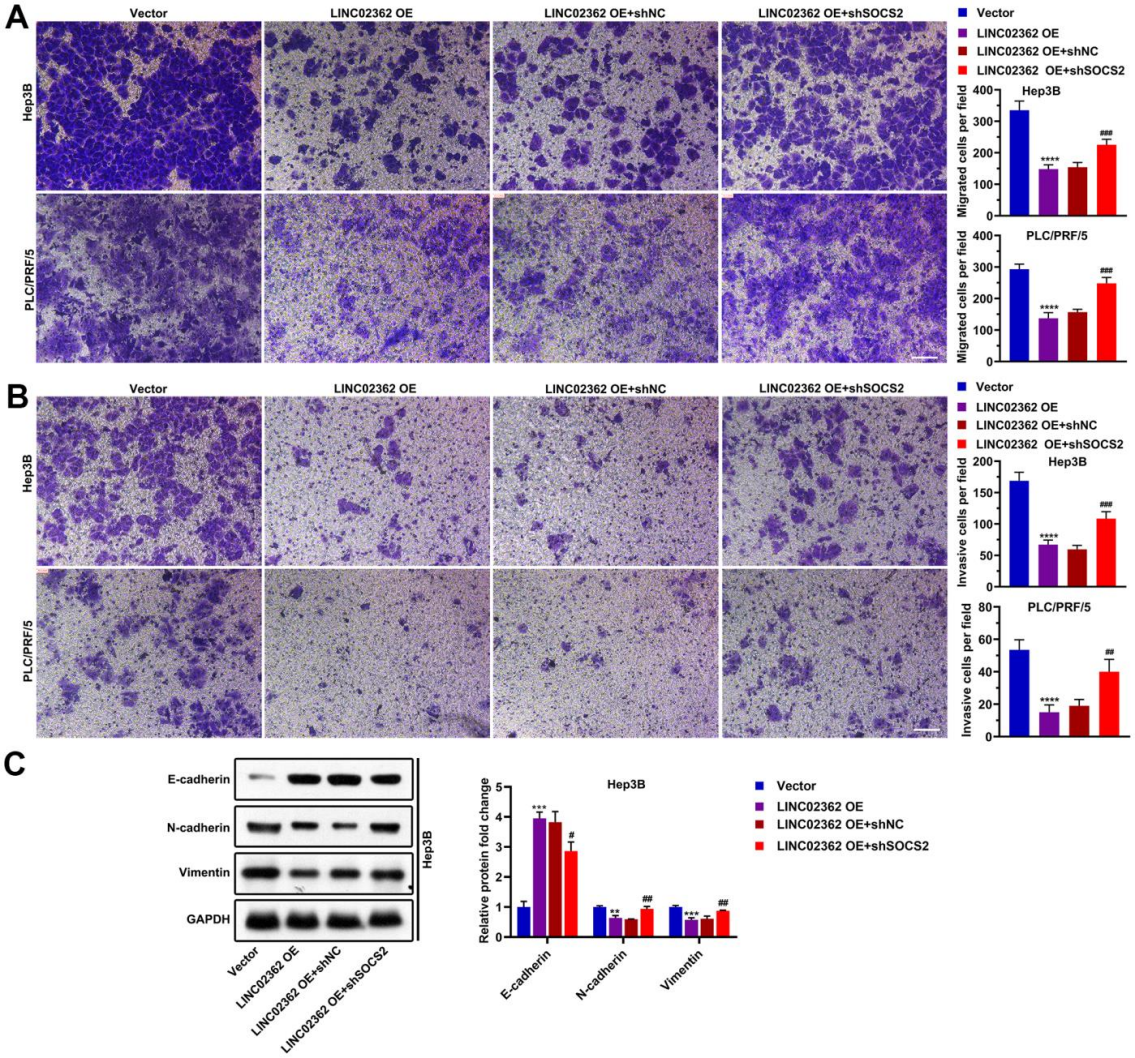
**SOCS2 is critical for the suppressive effects of *LINC02362* on HCC cell migration, invasion and EMT.** (**A**) Transwell assays for testing the effects of *LINC02362* and SOCS2 misexpression on HCC cell migration. Representative pictures (left) and quantification (right; n=3) are shown. (**B**) Transwell assays for testing the effects of *LINC02362* and SOCS2 misexpression on HCC cell invasion. Representative pictures (left) and quantification (right; n=3) are shown. (**C**) Representative images (left) and quantification (right; n=3) of western blotting analysis for detecting the levels of EMT markers in Hep3B cells. ## 0.001 < *P* < 0.01, *** or ### 0.0001 < *P* < 0.001, **** *P* < 0.0001.

To determine whether *SOCS2* is a key indirect target of *LINC02362* during the mitigation of HCC progression, we performed a series of rescue experiments. Results from RT-qPCR indicated that knockdown of *SOCS2* did not change the expression of *LINC02362*, suggesting that *LINC02362* could not be regulated by *SOCS2* (Figure 10A). However, when *SOCS2* was depleted, the inhibitory effects of *LINC02362* on HCC cell proliferation and cell cycle progression were attenuated, as demonstrated by MTT and EdU staining (Figure 10B–10H). On the contrary, the promotion of cell apoptosis by *LINC02362* was rescued upon the knockdown of *SOCS2* (Figure 10I, 10J). Moreover, *SOCS2* depletion also mitigated the suppressive effects of *LINC02362* on HCC cell migration, invasion and EMT (Figure 11A–11C). In conclusion, our data revealed that *SOCS2* is an indirect downstream target gene of *LINC02362* that inhibits HCC progression.

## DISCUSSION

**Figure 12:**
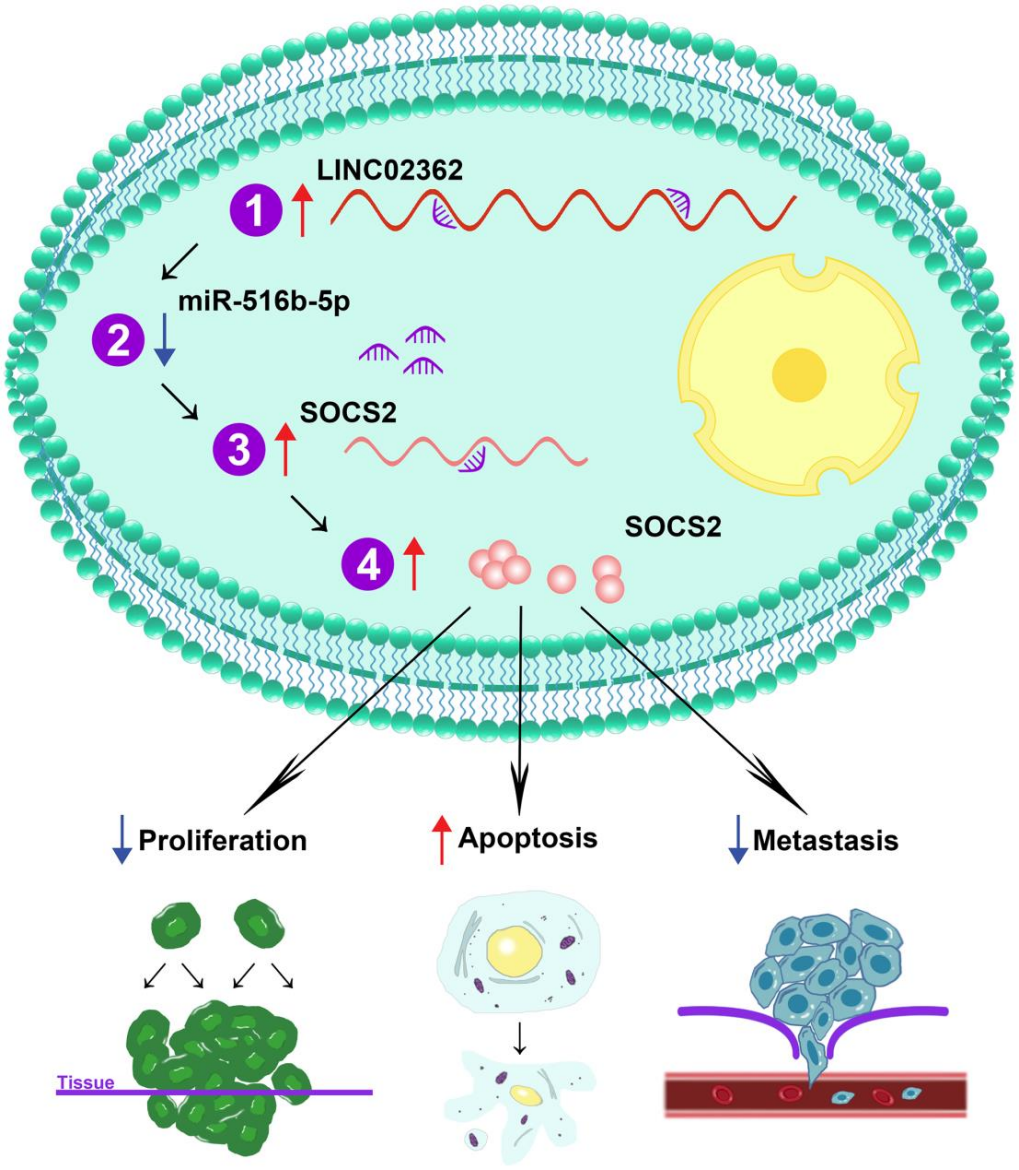
**Schematic working model of *LINC02362*/*miR-516b-5p*/SOCS2 axis on HCC progression.** Schematic model showing the mechanism how the *LINC02362*/*miR-516b-5p*/SOCS2 axis regulate the HCC progression. Upon the upregulation of *LINC02362*, downstream target miRNA *miR-516b-5p* is sponged and the levels are decreased in the HCC cells. As *miR-516b-5p* is a negative regulator of SOCS2, the *SOCS2* mRNA and protein levels are enhanced by *LINC02362*, resulting in the mitigation of cell proliferation and metastasis and augment of cell apoptosis of HCC cells.

In the present study, we unveil *LINC02362* as a novel tumor-inhibitory lncRNA by directly sponging *miR-516b-5p* and indirectly increasing the levels of *SOCS2*, leading to the attenuation of proliferation and metastasis as well as the augment of apoptosis in HCC cells (Figure 12). Datamining analyses are first exploited to assess the clinical significance of *LINC02362* in HCC. In Figure 1E, we find that the levels of *LINC02362* are upregulated in Child-Pugh grade C subgroup compared with stage A and B, which seems to be conflicted with our hypothesis. However, this might be because that only one patient is included in the grade C subclass, which cannot provide strong evidence of the clinical significance of *LINC02362*. To better understand the clinical value of *LINC02362*, we will generate our own cohort of HCC patients and apply the clinical information for further checking the correlation between *LINC02362* and HCC patient survival to determine whether it can be used as a prognostic biomarker.

The clinical significance of *LINC02362* is demonstrated in details by data mining, which provides an ideal candidate lncRNA for HCC prognosis. However, further investigations are required to better understand whether this lncRNA is eligible to serve as a biomarker. For example, it is of great significance to study the prognostic value of *LINC02362* in different subgroups of HCC in order to get more hints on which subset of patients can benefit from the therapeutic applications based on *LINC02362*.

*LINC02362* is a 766 nt lncRNA whose biological function has not been well-investigated. Here, we confirmed its cytoplasmic localization and the ceRNA mechanism. Many studies have shown that the interacting proteins are pivotal partners of one specific lncRNA in the regulation of cellular processes as well [23–26]. For instance, *NKILA* was proved to interact with the NF-κB complex in the cytosol to regulate the progression of breast cancer cells [23]. To fully mine the partners of *LINC02362*, systematic and unbiased approaches are required. For example, mass spectrum analysis can be exploited to screen the interacting proteins of *LINC02362* upon the RNA pull-down by *LINC02362* probes. In addition, RNA sequencing can be applied to uncover the downstream genes of *LINC02362*, which can be further used to enrich the pathways modulated by *LINC02362*.

Importantly, despite one lncRNA may have multiple miRNA targets, we enrich *miR-516b-5p* as a target miRNA of *LINC02362* via an unbiased method in which we take three independent databases into consideration. Although no significant association is observed between *miR-516b-5p* and *LINC02362*, there is indeed an inverse correlation trend between these two molecules (Figure 4G). Moreover, we validate that *miR-516b-5p* is a tumor-supporting miRNA in Figures 5, 6. To further ask the correlation between *miR-516b-5p* and *LINC02362*, it is worthy to test their expression in more HCC samples and preferably in our own cohort. Moreover, although the tumor-suppressive effects of *LINC02362* and the tumor-promoting effects of *miR-516b-5p* were determined by *in vitro* assays, we believe that extra results from *in vivo* models such as mice xenograft models may further confirm our conclusion.

We show that *LINC02362* inhibits HCC progression via enhancing the level of *SOCS2*, which is accomplished by sponging *miR-516b-5p*. SOCS2 has been proved to act as a pivotal tumor suppressor in various types of cancer. SOCS2 was confirmed to suppresses HCC cell proliferation, migration, and stemness recently [27]. However, the expression pattern and biological functions of *SOCS2* remains investigated. In this study, we link the underlying mechanism of *LINC02362* and *miR-516-5p* with *SOCS2*, which adds another layer of regulation to the alteration of *SOCS2* expression.

In summary, we identify *LINC02362* as an HCC-suppressive lncRNA that may be developed as a biomarker for HCC prognosis and a therapeutic agent. The *miR-516b-5p*/*SOCS2* axis is uncovered as the mechanism by which *LINC02362* regulate HCC progression, which offers a paradigm for investigating the regulatory mechanisms of lncRNAs in biological processes.

## MATERIALS AND METHODS

### Datamining

RNA sequencing data of HCC patients (n=369) and non-tumor tissues (n=50) were downloaded from the TCGA-LIHC database (https://portal.gdc.cancer.gov/). The clinical survival data, parameters, and mRNA and miRNA sequencing data of HCC patients were obtained from the UCSC Xena platform (https://tcga.xenahubs.net). The normalization of all sequencing data and differentially expressed genes were analyzed by R edgeR package (version 3.30.3). The threshold was |log2FC(fold change)| > 1 and FDR < 0.05. Patient survival analyses were performed using the Kaplan-Meier curve R survival package (Version 3.1 12). The p value was calculated by Log Rand test.

### Cell culture

HEK293T, Hep3B, and PLC/PRF/5 cells were purchased from the American Type Culture Collection (ATCC). All the cells were maintained in Dulbecco’s modified Eagle’s medium (DMEM; Thermo Fisher; 41966029) supplemented with 10% fetal bovine serum (FBS; Thermo Fisher; 26140079) and kept in a humidified incubator with 5% CO_2_ at 37° C, and were routinely checked for mycoplasma free.

### Real-time quantitative PCR (RT-qPCR)

Total RNA was extracted using TRIzol reagent (Invitrogen; 15596026). After measuring the RNA levels, the same amount of RNA (100 ng-1 μg) was reversely transcribed into cDNA using the RevertAid First Strand cDNA Synthesis Kit (Thermo Fisher; K1621). Subsequently, cDNAs were diluted five times with H_2_O and applied for qPCR together with the corresponding primers and SYBR Green PCR Master Mix (Thermo Fisher; 4309155) in the CFX-96 machine (Bio-Rad). All primers used in this study are listed in Supplementary Table 1. *18S* RNA was set as a reference transcript to calibrate the relative expression of each gene according to the 2 ^-ΔΔCt^ formula.

### 5-ethnyl-2 deoxyuridine (EdU) incorporation assays

EdU experiments were performed by applying an EdU Apollo 488 kit (RiboBio; C10310-1). Briefly, cells were seeded in wells of 24-well plate. The next day, cells were treated with EdU (50 μM) and kept in the incubator for 4 h. Subsequently, cells were fixed and treated with glycine, followed by permeabilization with 0.5% Triton X-100 in PBS for 5 min, and stained according to the manufacturer’s instructions.

### Lentiviral infection and transfection

Plasmids encoding cDNAs for shRNAs were transfected with the third generation of the packaging system (VSV, gag, and Rev) into HEK293T cells by the PEI solution (Sigma; 49553-93-7). The medium was changed after 24 h of transfection and collected at 48 h post-transfection. The virus-containing medium was filtered and added to target cells in 10% density at a 1:1 ratio with a fresh medium. Cells were changed into the fresh medium after 24 h of infection and subjected to puromycin selection (1 μg/mL) for 3-4 days. For transfection, Lipofectamine 300 (Thermo Fisher; L3000008) was used according to the manufacturer’s instructions. RNA and protein were collected 2 days after transfection.

### Flow cytometry analyses

For cell cycle analyses, 70% ethanol was applied to fix cells at 4° C overnight. Cells were then washed and treated with propidium iodide (Sigma; P4170; 50 μg/mL) and RNase A (Thermo Fisher; R1253) for 30 min at 37° C. Finally, at least 1 × 10^4^ cells were captured using a flow cytometer (BD Biosciences). For apoptosis detection, the Annexin V-FITC/PI Apoptosis Detection Kit (Sigma; APOAF-60TST). The signal cells were collected and washed twice with PBS. Afterwards, cells were then stained with Annexin V-FITC and PI according to the manufacturer’s instructions for 15 min at RT in the dark. The aforementioned flow cytometer was used to capture the signals from cells, and all data were quantified using the Flowjo software.

### Transwell assays

Cell migration and invasion were performed based on the Corning® Transwell® polycarbonate membrane cell culture inserts with 8.0 μm pore size (Merck; CLS3422-48EA). Briefly, 5 × 10^4^ Hep3B and PLC/PRF/5 cells were seeded in the top insert after 48 h of transfection with the indicated plasmids or miRNA mimics. At 48 h post-seeding, cells on the top flat were removed using a swab, while cells on the bottom flat were fixed with 4% PFA and stained with 0.1% crystal violet. Stained cells were photographed and counted under a microscope.

### Subcellular fractionation

Hep3B or PLC/PRF/5 cells (1 × 10^6^ cells) were collected after trypsinization. Subsequently, nuclear or cytosolic compartments were obtained using the PARIS™ Kit (Thermo Fisher; AM1921) according to the manufacturer’s instructions. The same amount of RNA was subjected to cDNA synthesis and RT-qPCR analyses.

### RNA fluorescent *in situ* hybridization (FISH)

To check the localization of circ0007360, we applied the RNA FISH method. The whole procedure was done based on the instructions from the Fluorescent *in situ* Hybridization Kit (Rabobio; C10910). The probes were also ordered from Rabobio as follows: FISH Probe Mix (Red; Rabobio; C10920), h-U6 FISH Probe Mix (Red) (Rabobio; LNC110101), h-18S FISH Probe Mix (Red; Rabobio; LNC110201).

### Dual luciferase reporter assays

Before the day of transfection, 1 x 10^5^ Hep3B or PLC/PRF/5 cells were seeded in wells of a 24-well plate. The next day, luciferase containing plasmids encoding the indicated 3’UTR regions or miRNA mimics were transfected into the cells using Lipofectamine 3000. Two days post-transfection, cells were lysed using passive lysis buffer, and the lysates were used to measure Firefly and Renilla luciferase activities using the Dual-Luciferase® Reporter Assay kit (Promega; E1910). Renilla luciferase activity was used for the normalization of relative luciferase activity.

### MTT analyses

MTT assays were performed to determine the viability of HCC cells. Briefly, 1 × 10^3^ Hep3B or PLC/PRF/5 cells were seeded in wells of 96-well plates in six replicates. At each time point, 10 μL MTT reagent (0.5 mg/ml at final concentration) was added to each well. After incubating the plates at 37° C for 4 h, the remaining medium was replaced with 100 μL DMSO to dissolve the cell contents. The absorbance values were measured using a plate reader at 490 nm wavelength.

### Western blotting

In brief, cells were lysed in RIPA buffer (Beyotime; P0013B) on ice for 20 min, followed by centrifugation. Total protein levels were quantified using a Pierce™ BCA Protein Assay Kit (Thermo Fisher; 23225). Next, the same amount of protein was separated by SDS-PAGE and transferred to a PVDF membrane. The membranes were then blocked with 5% milk in TBST, followed by incubation with primary antibodies (Supplementary Table 2) at 4° C overnight. After washing with TBST, the blots were incubated with the corresponding secondary antibodies. Finally, ECL substrate (Thermo Fisher; 32209) was used to expose the blots, and the films were subjected to signal capture.

### Statistical analyses

All data are expressed as the mean ± standard deviation. Student’s t test was used to analyze the statistical significance using GraphPad software. *P* < 0.05 was considered statistically significant (*0.01 < *P* < 0.05, **0.001 < *P* < 0.01, ***0.0001 < *P* < 0.001, ****P < 0.0001). NS, not significant.

## Supplementary Material

Supplementary Figures

Supplementary Tables

## References

[1] Villanueva A. Hepatocellular Carcinoma. N Engl J Med. 2019; 380:1450–62. 10.1056/NEJMra1713263 30970190

[2] Craig AJ, von Felden J, Garcia-Lezana T, Sarcognato S, Villanueva A. Tumour evolution in hepatocellular carcinoma. Nat Rev Gastroenterol Hepatol. 2020; 17:139–52. 10.1038/s41575-019-0229-4 31792430

[3] Hombach S, Kretz M. Non-coding RNAs: Classification, Biology and Functioning. Adv Exp Med Biol. 2016; 937:3–17. 10.1007/978-3-319-42059-2_1 27573892

[4] Batista PJ, Chang HY. Long noncoding RNAs: cellular address codes in development and disease. Cell. 2013; 152:1298–307. 10.1016/j.cell.2013.02.012 23498938 PMC3651923

[5] Schmitt AM, Chang HY. Long Noncoding RNAs in Cancer Pathways. Cancer Cell. 2016; 29:452–63. 10.1016/j.ccell.2016.03.010 27070700 PMC4831138

[6] Lin C, Yang L. Long Noncoding RNA in Cancer: Wiring Signaling Circuitry. Trends Cell Biol. 2018; 28:287–301. 10.1016/j.tcb.2017.11.008 29274663 PMC5869122

[7] Kondo Y, Shinjo K, Katsushima K. Long non-coding RNAs as an epigenetic regulator in human cancers. Cancer Sci. 2017; 108:1927–33. 10.1111/cas.13342 28776911 PMC5623749

[8] Wang Y, Chen F, Zhao M, Yang Z, Li J, Zhang S, Zhang W, Ye L, Zhang X. The long noncoding RNA HULC promotes liver cancer by increasing the expression of the HMGA2 oncogene via sequestration of the microRNA-186. J Biol Chem. 2017; 292:15395–407. 10.1074/jbc.M117.783738 28765279 PMC5602398

[9] Adams BD, Kasinski AL, Slack FJ. Aberrant regulation and function of microRNAs in cancer. Curr Biol. 2014; 24:R762–76. 10.1016/j.cub.2014.06.043 25137592 PMC4177046

[10] Yuan JH, Yang F, Wang F, Ma JZ, Guo YJ, Tao QF, Liu F, Pan W, Wang TT, Zhou CC, Wang SB, Wang YZ, Yang Y, et al. A long noncoding RNA activated by TGF-β promotes the invasion-metastasis cascade in hepatocellular carcinoma. Cancer Cell. 2014; 25:666–81. 10.1016/j.ccr.2014.03.010 24768205

[11] Xu D, Yang F, Yuan JH, Zhang L, Bi HS, Zhou CC, Liu F, Wang F, Sun SH. Long noncoding RNAs associated with liver regeneration 1 accelerates hepatocyte proliferation during liver regeneration by activating Wnt/β-catenin signaling. Hepatology. 2013; 58:739–51. 10.1002/hep.26361 23483581

[12] Wang Y, Zhu P, Wang J, Zhu X, Luo J, Meng S, Wu J, Ye B, He L, Du Y, He L, Chen R, Tian Y, Fan Z. Long noncoding RNA lncHand2 promotes liver repopulation via c-Met signaling. J Hepatol. 2018; 69:861–72. 10.1016/j.jhep.2018.03.029 29653123

[13] Letellier E, Haan S. SOCS2: physiological and pathological functions. Front Biosci (Elite Ed). 2016; 8:189–204. 10.2741/E760 26709655

[14] Qiu X, Zheng J, Guo X, Gao X, Liu H, Tu Y, Zhang Y. Reduced expression of SOCS2 and SOCS6 in hepatocellular carcinoma correlates with aggressive tumor progression and poor prognosis. Mol Cell Biochem. 2013; 378:99–106. 10.1007/s11010-013-1599-5 23475171

[15] Huang Z, Zhou JK, Peng Y, He W, Huang C. The role of long noncoding RNAs in hepatocellular carcinoma. Mol Cancer. 2020; 19:77. 10.1186/s12943-020-01188-4 32295598 PMC7161154

[16] Huang X, Gao Y, Qin J, Lu S. lncRNA MIAT promotes proliferation and invasion of HCC cells via sponging miR-214. Am J Physiol Gastrointest Liver Physiol. 2018; 314:G559–G565. 10.1152/ajpgi.00242.2017 29097358

[17] Özdemir F, Baskiran A. The Importance of AFP in Liver Transplantation for HCC. J Gastrointest Cancer. 2020; 51:1127–32. 10.1007/s12029-020-00486-w 32845425

[18] Erstad DJ, Tanabe KK. Prognostic and Therapeutic Implications of Microvascular Invasion in Hepatocellular Carcinoma. Ann Surg Oncol. 2019; 26:1474–93. 10.1245/s10434-019-07227-9 30788629

[19] Pastushenko I, Blanpain C. EMT Transition States during Tumor Progression and Metastasis. Trends Cell Biol. 2019; 29:212–26. 10.1016/j.tcb.2018.12.001 30594349

[20] Statello L, Guo CJ, Chen LL, Huarte M. Gene regulation by long non-coding RNAs and its biological functions. Nat Rev Mol Cell Biol. 2021; 22:96–118. 10.1038/s41580-020-00315-9 33353982 PMC7754182

[21] Rashid F, Shah A, Shan G. Long Non-coding RNAs in the Cytoplasm. Genomics Proteomics Bioinformatics. 2016; 14:73–80. 10.1016/j.gpb.2016.03.005 27163185 PMC4880952

[22] Pasquinelli AE. MicroRNAs and their targets: recognition, regulation and an emerging reciprocal relationship. Nat Rev Genet. 2012; 13:271–82. 10.1038/nrg3162 22411466

[23] Liu B, Sun L, Liu Q, Gong C, Yao Y, Lv X, Lin L, Yao H, Su F, Li D, Zeng M, Song E. A cytoplasmic NF-κB interacting long noncoding RNA blocks IκB phosphorylation and suppresses breast cancer metastasis. Cancer Cell. 2015; 27:370–81. 10.1016/j.ccell.2015.02.004 25759022

[24] Hosono Y, Niknafs YS, Prensner JR, Iyer MK, Dhanasekaran SM, Mehra R, Pitchiaya S, Tien J, Escara-Wilke J, Poliakov A, Chu SC, Saleh S, Sankar K, et al. Oncogenic Role of THOR, a Conserved Cancer/Testis Long Non-coding RNA. Cell. 2017; 171:1559–72.e20. 10.1016/j.cell.2017.11.040 29245011 PMC5734106

[25] Grelet S, Link LA, Howley B, Obellianne C, Palanisamy V, Gangaraju VK, Diehl JA, Howe PH. A regulated PNUTS mRNA to lncRNA splice switch mediates EMT and tumour progression. Nat Cell Biol. 2017; 19:1105–15. 10.1038/ncb3595 28825698 PMC5578890

[26] Leucci E, Vendramin R, Spinazzi M, Laurette P, Fiers M, Wouters J, Radaelli E, Eyckerman S, Leonelli C, Vanderheyden K, Rogiers A, Hermans E, Baatsen P, et al. Melanoma addiction to the long non-coding RNA SAMMSON. Nature. 2016; 531:518–22. 10.1038/nature17161 27008969

[27] Cui M, Sun J, Hou J, Fang T, Wang X, Ge C, Zhao F, Chen T, Xie H, Cui Y, Yao M, Li J, Li H. The suppressor of cytokine signaling 2 (SOCS2) inhibits tumor metastasis in hepatocellular carcinoma. Tumour Biol. 2016; 37:13521–31. 10.1007/s13277-016-5215-7 27465557

