# Supplementary material for: LINC02362 attenuates hepatocellular carcinoma progression through the miR-516b-5p/SOSC2 axis
Source: Aging (Albany NY). 2022 Jan 6;14(1):368–88. doi: 10.18632/aging.203813 (PMC8791201; doi:10.18632/aging.203813)
Supplement: Supplementary Figures [file aging-14-203813-s001.pdf]

SUPPLEMENTARY FIGURES

------------ lncLocator Prediction Result ------------

Subcellular locations	score
Cytoplasm	0.844761316248
Nucleus	0.0188670440491
Ribosome	0.0191748094148
Cytosol	0.1153390012
Exosome	0.00185782908802

Predicted location
Cytoplasm

Supplementary Figure 1. *LINC02362* is mainly localized in the cytoplasm by database mining. Results from lncLocator (http://www.csbio.sjtu.edu.cn/bioinf/lncLocator/) for predicting the localization of *LINC02362*.

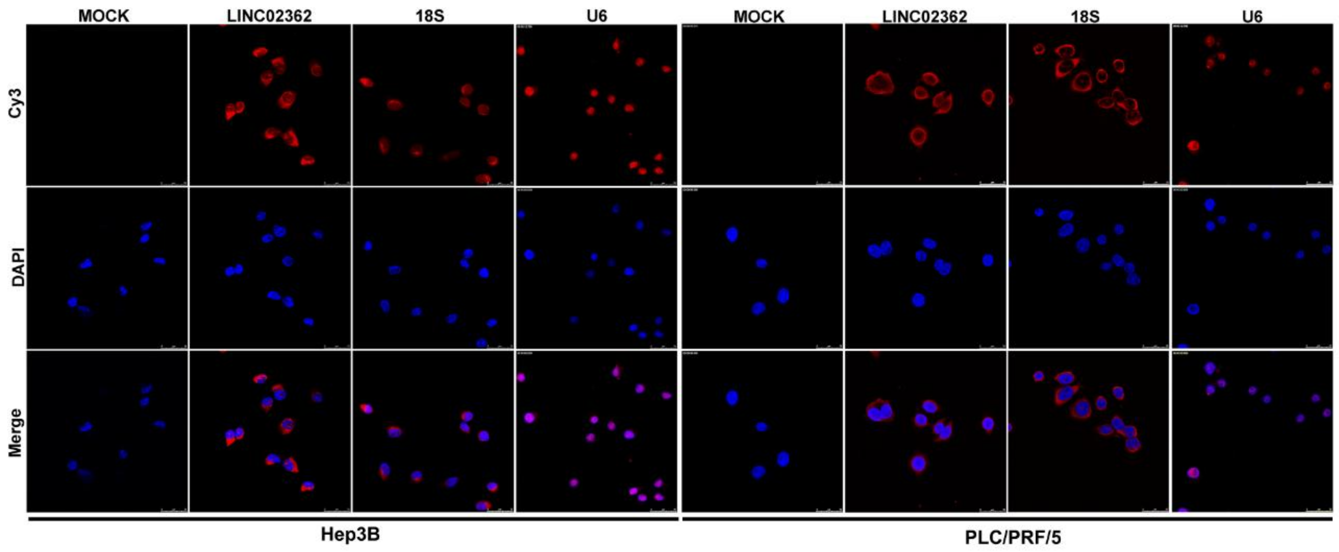

Supplementary Figure 2. *LINC02362* is localized in the cytoplasm. Representative images from RNA fluorescent *in situ* hybridization (FISH) for detecting *LINC02362*, 18S or U6 RNA. DAPI was applied to stain the nucleus.
