# Supplementary material for: LINC02362 attenuates hepatocellular carcinoma progression through the miR-516b-5p/SOSC2 axis
Source: Aging (Albany NY). 2022 Jan 6;14(1):368–88. doi: 10.18632/aging.203813 (PMC8791201; doi:10.18632/aging.203813)
Supplement: Supplementary Tables [file aging-14-203813-s002.pdf]

SUPPLEMENTARY TABLES

Supplementary Table 1. Primers for plasmid construction and RT-qPCR.

Primers		Sequence (5'-3')
ShSOCS2	F	CCGGCATTCAGACTACCTACTAACACTCGAGTGTTAGTAGGTAGTCTGAATGTTTTT
	R	AATTAAAAACATTCAGACTACCTACTAACACTCGAGTGTTAGTAGGTAGTCTGAATG
Sh*LINC02362*	F	CCGGGGATGTCTGTCACACCTCAGGCTCGAGCCTGAGGTGTGACAGACATCCTTTTT
	R	AATTAAAAAGGATGTCTGTCACACCTCAGGCTCGAGCCTGAGGTGTGACAGACATCC
U6	RT	AACGCTTCACGAATTTGCGT
	QF	CTCGCTTCGGCAGCACA
	QR	AACGCTTCACGAATTTGCGT
miR-516b-5p	RT	GTCGTATCCAGTGCAGGGTCCGAGGTATTCGCACTGGATACGACAAAGTG
	QF	GCGCGATCTGGAGGTAAGAAG
	QR	AGTGCAGGGTCCGAGGTATT
18S	F	AGGCGCGCAAATTACCCAATCC
	R	GCCCTCCAATTGTTCCTCGTTAAG
SOCS2	F	GGTCGGCGGAGGAGCCATCC
	R	GAAAGTTCCTTCTGGTGCCTCTT
LINC02362	F	TGGAGGATACTGGTCTGA
	R	TGCTGCCTTATCTGGAAT
mimic NC	F	UUCUCCGAACGUGUCACGUTT
	R	ACGUGACACGUUCGGAGAATT
miR-516b-5p mimic	F	AGUGCUUCUUACCUCCAGAUUU
	R	AUCUGGAGGUAAGAAGCACUUU
Inhibitor NC		CAGUACUUUUGUGUAGUACAA
miR-516b-5p inhibitor		AGUGCUUCUUACCUCCAGAUUU

Supplementary Table 2. Antibodies for western blotting.

Antibodies	Provider	Catalog no.	Dilution
GAPDH	Proteintech, Wuhan, China	10494-1-AP	1:3000
SOCS2	Abcam, Shanghai, China	ab109245	1:2000
E-cadherin	Proteintech, Wuhan, China	20874-1-AP	1:6000
N-cadherin	Proteintech, Wuhan, China	22018-1-AP	1:3000
Vimentin	Proteintech, Wuhan, China	10366-1-AP	1:3000
HRP Goat Anti-mouse IgG(H+L)	Proteintech, Wuhan, China	SA00001-1	1:10000
HRP Goat Anti-Rabbit IgG(H+L)	Proteintech, Wuhan, China	SA00001-2	1:10000

Supplementary Table 3. Correlation between clinicopathological variables and LINC02362 expression in HCC.

	Total (N=339)	LINC02362 Expression High (N=144)	Low (N=195)	P-value
**Age (year)**				
< 65	208 (61.4%)	76 (52.8%)	132 (67.7%)	***0.0074***
≥ 65	131 (38.6%)	68 (47.2%)	63 (32.3%)	
**Gender**				
Male	231 (68.1%)	109 (75.7%)	122 (62.6%)	***0.0144***
Female	108 (31.9%)	35 (24.3%)	73 (37.4%)	

**Family history of cancer**				
NO	196 (57.8%)	72 (50.0%)	124 (63.6%)	***0.0362***
YES	98 (28.9%)	51 (35.4%)	47 (24.1%)	
Unknown	45 (13.3%)	21 (14.6%)	24 (12.3%)	
**TNM stage**				
I	170 (50.1%)	81 (56.2%)	89 (45.6%)	0.182
II	84 (24.8%)	34 (23.6%)	50 (25.6%)	
III	81 (23.9%)	27 (18.8%)	54 (27.7%)	
IV	4 (1.2%)	2 (1.4%)	2 (1.0%)	
**Histologic grade**				
G1–G2	212 (62.5%)	105 (72.9%)	107 (54.9%)	***0.0027***
G3–G4	125 (36.9%)	38 (26.4%)	87 (44.6%)	
Unknown	2 (0.6%)	1 (0.7%)	1 (0.5%)	
**Ishak score**				
0-4	124 (36.6%)	63 (43.8%)	61 (31.3%)	***0.0038***
5-6	74 (21.8%)	36 (25.0%)	38 (19.5%)	
Unknown	141 (41.6%)	45 (31.2%)	96 (49.2%)	
**Child–Pugh grade**				
A	207 (61.1%)	98 (68.1%)	109 (55.9%)	0.0722
B-C	21 (6.2%)	8 (5.6%)	13 (6.7%)	
Unknown	111 (32.7%)	38 (26.4%)	73 (37.4%)	
**Vascular invasion**				
None	193 (56.9%)	94 (65.3%)	99 (50.8%)	***0.0429***
Micro	84 (24.8%)	30 (20.8%)	54 (27.7%)	
Macro	14 (4.1%)	3 (2.1%)	11 (5.6%)	
Unknown	48 (14.2%)	17 (11.8%)	31 (15.9%)	
**Alpha fetoprotein**				
Negative	143 (42.2%)	86 (59.7%)	57 (29.2%)	***<0.001***
Positive	120 (35.4%)	30 (20.8%)	90 (46.2%)	
Unknown	76 (22.4%)	28 (19.4%)	48 (24.6%)	
**Residual tumor**				
R0	301 (88.8%)	127 (88.2%)	174 (89.2%)	0.924
R1-R2	12 (3.5%)	5 (3.5%)	7 (3.6%)	
Unknown	26 (7.7%)	12 (8.3%)	14 (7.2%)	
**Living status**				
Alive	224 (66.1%)	106 (73.6%)	118 (60.5%)	***0.0163***
Dead	115 (33.9%)	38 (26.4%)	77 (39.5%)	
**Disease status**				
NO	163 (48.1%)	65 (45.1%)	98 (50.3%)	0.536
YES	132 (38.9%)	61 (42.4%)	71 (36.4%)	
Unknown	44 (13.0%)	18 (12.5%)	26 (13.3%)	
